# Denosumab improves bone mineral density and microarchitecture and reduces bone pain in women with osteoporosis with and without glucocorticoid treatment

**DOI:** 10.1080/13102818.2014.967827

**Published:** 2014-10-17

**Authors:** Tzvetanka Petranova, Ivan Sheytanov, Simeon Monov, Rodina Nestorova, Rasho Rashkov

**Affiliations:** ^a^ Clinic of Rheumatology, Medical University, Sofia, Bulgaria; ^b^ Rheumatology Center ‘Sveta Irina’, Sofia, Bulgaria

**Keywords:** denosumab, postmenopausal osteoporosis, glucocorticoid treatment, BMD, TBS, bone pain

## Abstract

Osteoporosis is a key health problem in postmenopausal women with high social and economic impact. Decreased bone mineral density (BMD) and deterioration of bone microarchitecture may occur also as a result of long-term glucocorticoid treatment (GCT) of autoimmune or inflammatory conditions. Denosumab specifically inhibits the binding of the receptor activator of nuclear factor-κB to its ligand, thus preventing osteoclast activation and bone resorption. The efficacy and safety of denosumab, administered subcutaneously as 60 mg, once every six months for 12 months, were evaluated in 60 patients with postmenopausal osteoporosis (PMO) divided into two groups. The GCT group included 30 patients receiving concomitant glucocorticoid therapy and the non-GCT group included 30 patients that did not receive GCT. In the non-GCT group, the 12-month treatment with denosumab resulted in BMD increase of 6.1% and 2.8% in lumbar spine and hip, respectively. T-score increased by 13.1% and 5.6% in both, the lumbar spine and hip. A slight rise in the Trabecular Bone Score (TBS) of 0.3% was observed. Bone pain was markedly reduced by 56.2%. In the GCT group, denosumab therapy increased BMD with 5.8% and 2.3% in lumbar spine and hip, respectively. T-score of lumbar spine and hip significantly increased by 14.0% and 4.4%, and the TBS rose by 5%. Bone pain was reduced by 53.6%. These data confirm the available knowledge on denosumab efficacy and safety in women with PMO and also provide new insights into its therapeutic potential in patients with osteoporosis related to a long-term corticosteroid treatment.

## Introduction

Osteoporosis (OP) is a public health problem representing a common bone disease characterized by bone loss and structural deterioration of bone tissue. OP can lead to numerous other clinical and health-related consequences, including fracture, the need for long-term care and excess mortality. The reduced bone density associated with the disorder is a major risk factor for fracture, especially of the hip, spine and wrist.[1] OP is often referred to as a silent disease, as many individuals do not realize that they have the disease until a fracture occurs. OP-related fractures impose a heavy burden on individuals and on society, as they often lead to a variety of physical and psychological consequences, including future fractures, depression, functional impairment, pain and disability.[2] In postmenopausal osteoporosis (PMO), receptor activator of nuclear factor-κB ligand (RANKL) is directly associated with osteoclast-mediated bone resorption.[3,4] Osteoprotegerin binds to RANKL and is directly involved in the regulation of the RANK-RANKL pathway.[5]

OP may occur as a secondary disease and is one of the most devastating side-effects of glucocorticoid (GC) use and is associated with substantial morbidity.[6] GCs are frequently used for the treatment of a variety of diseases, such as rheumatoid arthritis, systemic lupus erythematosus, polymyalgia rheumatica, inflammatory bowel disease and chronic obstructive pulmonary disease. They are widely used because of their anti-inflammatory and immunosuppressive effects. Bone loss related to a long-term corticoid treatment is associated with elevated fracture incidence. It is estimated that fractures occur in 30%–50% of patients receiving a long-term GC therapy, which may have a substantial negative impact on the quality of life.[7]

Denosumab (Prolia®) is indicated for treatment of OP in postmenopausal women at increased risk of fractures. Prolia® significantly reduces the risk of vertebral, non-vertebral and hip fractures.[8] The mechanism of action of denosumab leads to rapid and maximal reductions in bone resorption throughout the trabecular and cortical compartments.[9] Denosumab is a fully human monoclonal antibody that potently blocks the binding of RANKL to its osteoclast-derived receptor (RANK), an interaction that is required for osteoclast formation, activation and survival. By blocking this receptor binding, denosumab potently inhibits osteoclast-mediated bone resorption. This mechanism differs from that of bisphosphonates, which act via inhibition of the enzyme farnesyl pyrophosphate synthase, leading to decreased osteoclast activity and increased osteoclast apoptosis.[10]

Denosumab has already been established as an attractive new therapeutic agent for patients with increased fracture risk due to long-term GC treatment with renal failure and for GC-treated patients with rheumatoid arthritis. In a 12-month, placebo-controlled trial in patients with rheumatoid arthritis concurrently receiving treatment with GCs or bisphosphonates, denosumab therapy was shown to increase bone mineral density (BMD) and reduce bone turnover markers.[11] It has been suggested that denosumab may be considered for GC-treated patients with renal insufficiency and stable serum calcium levels who are not candidates for bisphosphonates or teriparatide. Moreover, the ease of administration as a subcutaneous injection every six months may increase patients’ compliance.[12]

The aim of this study was to evaluate the efficacy and safety of denosumab treatment on BMD, Trabecular Bone Score (TBS), T-score, Fracture Risk Assessment Tool (FRAX) and bone pain in patients with PMO with or without corticoid treatment.

## Materials and methods

### Subjects

Patients were divided into two groups: non-GCT group – women with PMO that did not receive GC therapy (*n* = 30); and GCT group – women with PMO receiving concomitant glucocorticoid treatment (GCT) (*n* = 30). All included patients had BMD score lower than –2.5 both at the lumbar spine and total hip and were treated with 60 mg denosumab *s.c.* once per six months, with Ca and vitamin D supplementation as per product indication.[8] In the non-GCT group, 23% of the patients had a concomitant rheumatology disease and 6.6% had an endocrine disease. Forty-seven per cent of the patients had received a previous OP treatment. Patients who had a previous osteoporotic fracture (either wrist, lumbar or proximal femur) represented 20% of this study group. With regard to the GCT group, 97% of the subjects had a concomitant rheumatology disease and 3% had an endocrine disease. Previous fractures had occurred among 50% of these patients (Table 1).

**Table 1.** Patients’ baseline characteristics and demographics.

#	Patients not treated with corticosteroids	Patients treated with corticosteroids
Female, *n* (%)	30 (100)	30 (100)
Race/ethnicity, *n* (%)		
White	30 (100)	30 (100)
Age (yr), mean (SD)	67.5 (9.0)	66.7 (7.9)
Age (yr) of menopause (SD)	47 (3.0)	48.2 (2.3)
Osteoporosis occurrence (yr)	63 (8.3)	61 (7.5)
Height (cm), mean (SD)	158 (5.9)	157 (5.7)
Weight (kg), mean (SD)	59 (14.1)	68 (12.7)
Smoking (Yes/No/Missing (%))	13/80/7	13/77/10
Family predisposition, fractures (Yes/No (%))	27/73	20/80
Rheumatology disease (Yes/No (%))	23/77	97/3
Endocrine disease (Yes/No (%))	6.6/93.4	3/97
Treatment with cortisone drug (%)	No (100)	Yes (100)
Administration of other medications (Yes/No (%))	20/80	90/10
Fractures, total number (% of pts)	0–3 (20)	0–4 (50)
Lumbar fractures	0–2	0–7
Wrist fractures	0–2	0–1
Fractures of proximal femur	0–1	0
Previous osteoporosis treatment (Yes/No (%))	46.6/53.4	53.3/46.6
Ca and vitamin D administration (Yes/NA (%))	43.3/56.7	70/30
Lumbar spine T-score, *n* (%)		
≤−2.5	27 (83)	24 (80)
>−2.5	3 (17)	6 (20)
Total hip T-score, *n* (%)		
≤−2.5	13 (43.3)	19 (63)
>−2.5	15 (50)	11 (37)
Missing	2 (6.7)	0
TBS, mean (SD)	1.292 (0.08)	1.168 (0.12)
Calcium (mmol/L), mean (SD)^§^	2.35 (0.1)	2.37 (0.1)
Phosphorus (mmol/L), mean (SD)^§^	1.17 (0.2)	1.13 (0.2)
Bone-specific alkaline phosphatase (U/l), mean (SD)^§^	90.8 (41.2)	88 (67)
Intact PTH (pg/ml), mean (SD)^§^	48.9 (14.9)	47.8 (14.3)
Beta-CTx, mean (SD)^§^	0.43 (0.3)	0.62 (1.4)
Osteocalcin^§^	24.8 (9.0)	16.4 (8.0)
Fractures after treatment initiation, *n* (%)	1 (3.3)	3 (10)

^§^Mean (SD) or reference range for normal values: 35 (15) nmol/mmol for urine NTX/creatinine (premenopausal women); 0.321 (0.155) ng/ml for serum CTx; 7.3–22.4 g/l for bone-specific alkaline phosphatase; 8.4–10.3 mg/dl for albumin-adjusted calcium; and 10–65 pg/ml for iPTH.

### DXA, BMD and TBS assessments

For all patients, BMD assessments were performed at baseline and after 12 months for the lumbar spine and total hip. BMD was measured by a Lunar Prodigy® Primo (GE Healthcare, Caserta, Italy) osteodensitometer. A new software program (TBS iNsight® v1.9, Med-Imaps, Pessac, France) was applied to the standard lumbar spine Dual-energy X-ray absorptiometry (DXA) scans to determine their TBS indexes. Lumbar spine scans included L1–L4 vertebrae. The scanner precision (precision error), together with the quality and reliability of individual scans, was measured using the LSC (least significant change) calculation. The precision error measured for the osteodensitometer used in the present work was 0.015 g/cm^2^ for posterior–anterior spine (LSC of 0.042 g/cm^2^) and 0.008 g/cm^2^ for the femoral neck (LSC of 0.022 g/cm^2^) which was close to the minimum acceptable precision for an individual technologist.[13]

### FRAX measurement and fracture risk assessment

The fracture risk was assessed for all patients in both groups (*n* = 30 subjects per group) using the online FRAX® tool at http://www.shef.ac.uk/FRAX/tool.jsp. At baseline, the estimated risk of major osteoporotic fracture and for hip fracture was 9.7% and 5.0% in the non-GCT group and 34% and 14% for the GCT group, respectively.

### Assessment of bone pain

Bone pain was evaluated by a visual analogue scale (VAS). Pain scores were assessed at baseline and after 12 months of treatment. Pain was measured using a 100-mm VAS with 0 representing ‘no pain’ and 100 representing ‘severe pain’.[14]

### Statistical analysis

**Figure 1.**
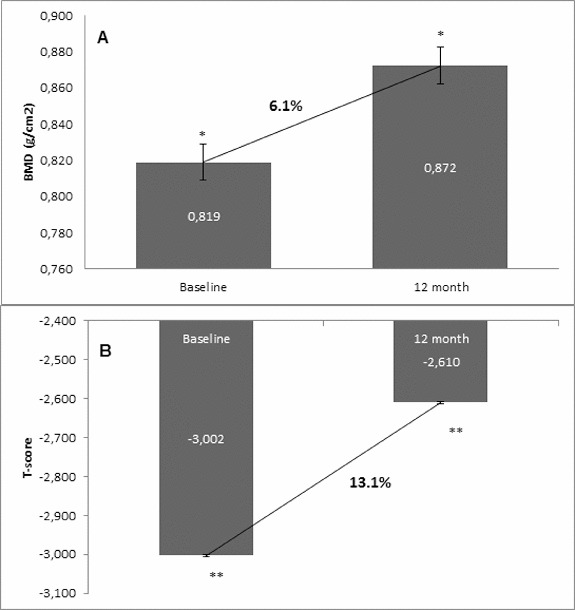
Change in BMD and T-score of lumbar spine after 12-month treatment with denosumab (non-GCT group). (A) Change in BMD. Data are presented as a comparison between mean values of BMD (g/cm^2^) at baseline and at the 12th month of treatment with a percent change of 6.1 and level of significance of *p* = 0.2. (B) Change in T-score. Comparison between mean values of T-score at baseline vs. T-score at month 12 of treatment with a significant change of 13.1%, *p* < 0.05.

**Figure 2.**
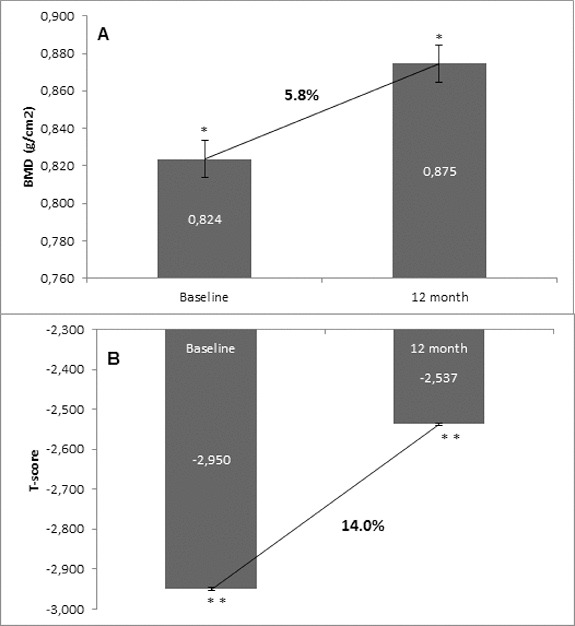
Change in BMD and T-score of lumbar spine after 12-month treatment with denosumab (GCT group). (A) Change in BMD. Data are presented as a comparison between mean values of BMD (g/cm^2^) at baseline and at the 12th month of treatment with a percent change of 5.8% and level of significance of *p* = 0.2. (B) Change in T-score. Comparison between mean values of T-score at baseline vs. T-score at month 12 of treatment with a significant change of 14.0%, *p* = 0.03.

**Figure 3.**
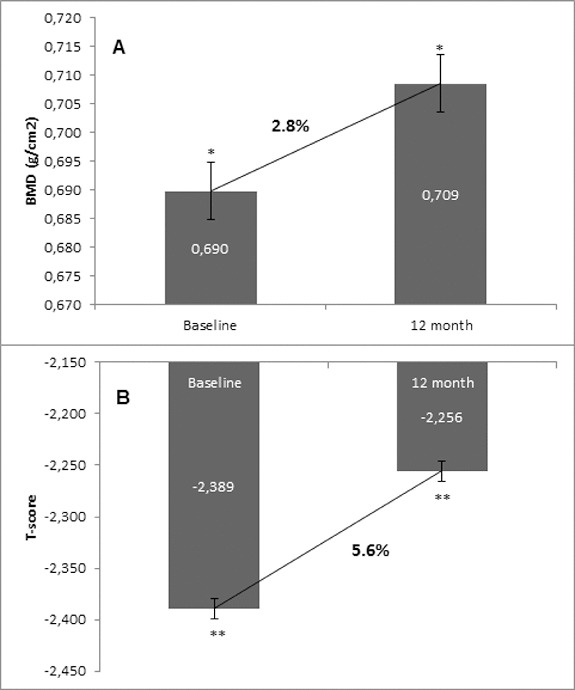
Change in BMD and T-score of total hip after 12-month treatment with denosumab (non-GCT group). (A) Change in BMD. Data are presented as a comparison between mean values of BMD (g/cm^2^) at baseline and at the 12th month of treatment with a percent change of 2.8% and level of significance of *p* = 0.2. (B) Change in T-score. Comparison between mean values of T-score at baseline vs. T-score at month 12 of treatment with a significant change of 5.6%, *p* = 0.01.

**Figure 4.**
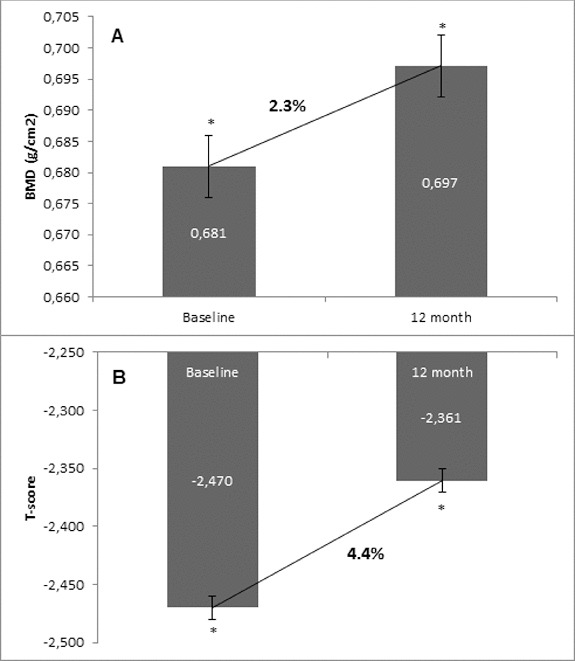
Change in BMD and T-score of total hip after 12-month treatment with denosumab (GCT group). (A) Change in BMD. Data are presented as a comparison between mean values of BMD (g/cm^2^) at baseline and at the 12th month of treatment with a percent change of 2.3% and level of significance of *p* = 0.2. (B) Change in T-score. Comparison between mean values of T-score at baseline vs. T-score at month 12 of treatment with a significant change of 4.4%, *p* = 0.01.

**Figure 5.**
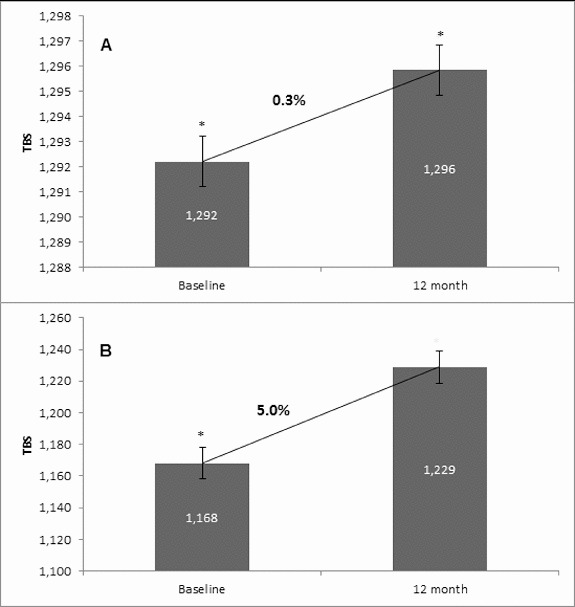
Effect of denosumab on TBS. (A) Non-GCT group. Comparison between mean TBS values at baseline and mean TBS values at the 12th month of denosumab therapy with a percent change of 0.3% and *p* = 0.1. (B) GCT group. Comparison between mean TBS values at baseline and mean TBS values at the 12th month of denosumab therapy with a percent change of 5.0% and *p* = 0.1.

**Figure 6.**
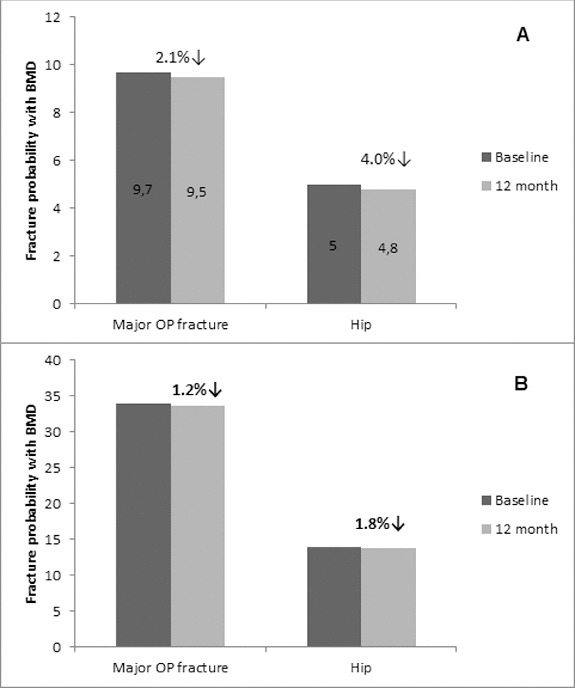
Effect of denosumab on fracture risk reduction. Data are presented as mean values (baseline and at the 12 month) of fracture risk for major osteoporotic and hip fracture, respectively, as measured by the FRAX tool. (A) Non-GCT group. (B) GCT group.

**Figure 7.**
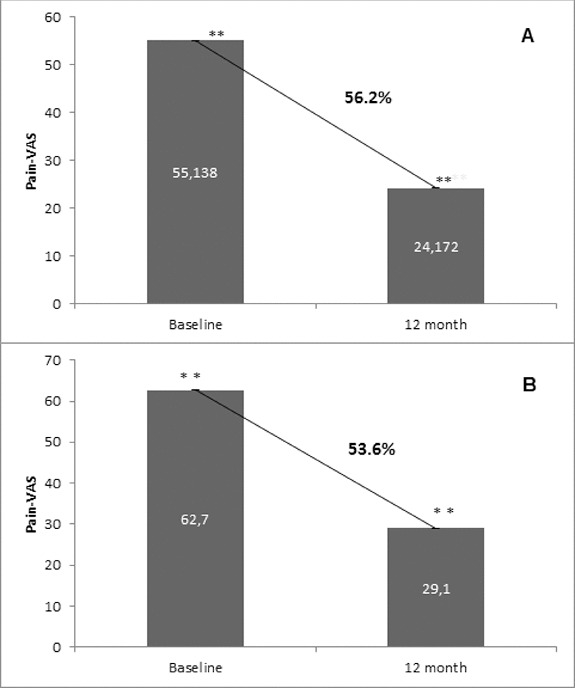
Effect of denosumab on bone pain. (A) Non-GCT group. Pain levels are presented as mean values of pain scores (VAS) assessed at baseline and after 12 months of treatment with a significant change of 56.2%, *p* < 0.01. (B) GCT group. Pain levels are presented as mean values of pain scores (VAS) assessed at baseline and after 12 months of treatment with a significant change of 53.6%, *p* < 0.01.

**Figure 8.**
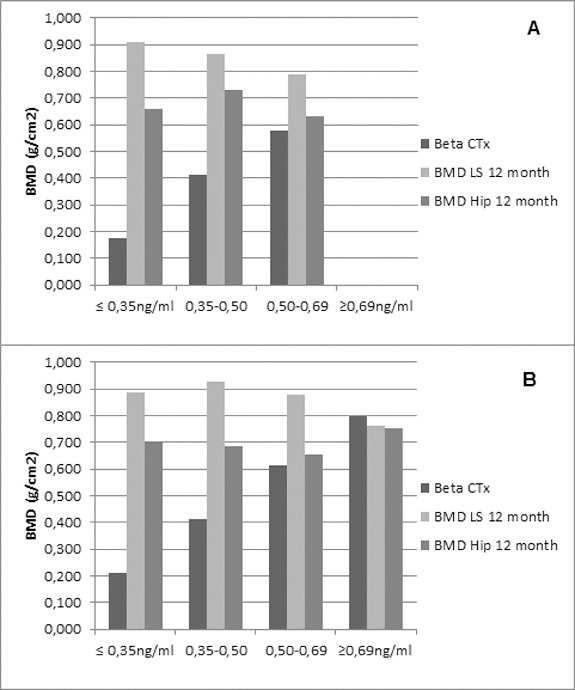
Correlation between BMD change (lumbar spine and hip) and Beta-CTx. Patients were divided into four groups on the basis of their baseline Beta-CTx measures. Data are presented as mean values of baseline Beta-CTx (per group) and mean BMD change (lumbar spine and hip, also per group) at month 12. (A) Non-GCT group. (B) GCT group.

The analysis was performed using the *T*-test for establishment of difference of mean values. Results were presented as mean statistical values of the analysed parameters: BMD; T-score; TBS; and values obtained from the VAS for assessing bone pain (comparison was made between the measurements at baseline and at month 12). Standard deviations and *p*-values are presented for each investigated parameter with a significance level of *p* ≤ 0.05. Percentage changes from baseline to the 12th month for all measured parameters are also presented (Figures 1–8).

## Results and discussion

OP is a common clinical condition, resulting in decreased bone strength, increased fracture risk and low quality of life. Currently, there is a great amount of evidence about the correlation between fracture risk and bone parameters such as BMD, T-score, TBS, bone turnover markers and bone pain. BMD is a key factor continuously used for demonstration of bone mass change in response to different anti-osteoporotic treatments. On the other hand, TBS reflects the status of bone microarchitecture.

### Changes of BMD and T-score of lumbar spine from baseline to month 12

In the non-GCT group, denosumab increased the BMD of lumbar spine at month 12 compared with the baseline BMD values in 93.3% of all patients. A percent change of 6.1% in BMD favouring denosumab treatment was measured at the end of observation. In correlation with the increase of BMD at the lumbar spine, the T-score was improved in 96.6% of patients, resulting in a significant change of 13.1% compared to the baseline T-score mean value (Figure 1). For the GCT group, BMD of lumbar spine was increased at month 12 after denosumab treatment in 96.6% of all patients compared with the baseline BMD values. A percent change of 5.8 in BMD favouring denosumab treatment was measured at the end of observation. The T-score was significantly improved in 96.6% of patients with 14.0% compared to the baseline T-score mean value (Figure 2).

The one-year clinical experience presented herein showed a clear trend for increase of BMD in the two groups that were managed: the GCT and non-GCT groups. Moreover, both groups showed a similar extent of the BMD increase despite bone localization, as well as between the levels of change. The results showed that in the non-GCT group, BMD of lumbar spine increased by 6.1% at month 12, and similarly, in the GCT group this percent change was 5.8%. BMD of total hip in the non-GCT and GCT groups increased up to month 12 by 2.8% and 2.3%, respectively. When the two groups were compared, a higher increase of BMD of lumbar spine than in the BMD of total hip was observed.

### Changes of BMD and T-score of total hip from baseline to month 12

In the non-GCT group, 22 patients (73.3%) had a BMD increase of total hip at month 12. There was a 2.8% increase in the BMD at the end of observation in comparison with the mean baseline values. Similarly to the results related to the T-score change at lumbar spine, the T-score of total hip also changed in corresponding proportion to the increase of BMD. There was a significant positive change of 5.6% in the T-score of total hip, determined in 80% of the patients as compared with their baseline T-score values (Figure 3).

In the GCT group, at month 12, 83.3% had a BMD increase of total hip. There was a 2.3% increase in the BMD at the end of observation in comparison with the mean baseline values. T-score of total hip also changed in corresponding proportion to the increase of BMD. There was a significant positive change of 4.4% in the T-score of total hip, determined in 83.3% of the patients as compared with their baseline T-score values (Figure 4).

Generally, T-score changed in a similar extent to BMD. In both groups, T-score showed a significant and constant increase. In the non-GCT group, at month 12, T-score of lumbar spine increased by 13.1% and in the GCT group, this increase was by 14.0%.

### Change in TBS

In the non-GCT group, the treatment of osteoporotic postmenopausal women with denosumab for 12 months led to an increase of TBS by 0.3%. For the one-year treatment period, this increase was documented in 70.0% of all patients. In the GCT group, TBS changed by 5.0% as compared to the baseline mean value. This change was observed in 76.6% of all patients (Figure 5). Interestingly, there was a distinct change in TBS between the two observed groups. This finding is in correlation with the concept that TBS provides distinct information, independent of BMD and can be used as an additional and substantive factor for assessing bone structure, quality and treatment effect.[15] Our results, together with the fact that TBS influences fracture risk, suggest that this novel technique may have a role in managing patients with OP.

### Fracture risk reduction in response to denosumab treatment at month 12

It was obvious that the patients from the GCT group had a higher risk of major osteoporotic and hip fracture at baseline (34% and 14%, respectively) as measured by the FRAX tool. It is most likely related to the long-term exposure of these patients to corticoid treatment. In opposite, the fracture risk reduction that was measured at the 12th month of denosumab treatment was lower in that group (1.2% of major OP fractures and 1.8% of hip fractures, respectively), showing a slower bone recovery in comparison to the non-GCT group which is in correlation with the data on BMD and T-score showing trends for increase. In the non-GCT group, the measured fracture risk at baseline as 9.7% and 5.0% for major OP and hip fracture, respectively, was reduced after 12 months of treatment with 2.1% and 4.0%, respectively. It is suggested that these reduction rates will increase significantly for a longer treatment period (Figure 6).

### Bone pain reduction

Denosumab treatment exhibited a substantial decrease in bone pain at month 12 as evaluated by the patients using VAS. This reduction was observed and reported by all patients from both the non-GCT and GCT groups with an average percent reduction of 56.2% and 53.6%, respectively (Figure 7).

### Markers of bone turnover

An additional analysis of the correlation between BMD changes (both for lumbar spine and hip) and Beta-CTx in the two groups was performed. It demonstrated that BMD increases and keeps equal levels of improvement despite the baseline value of Beta-CTx (Figure 8). The current results are in accordance with already published data on denosumab efficacy on the increase of BMD in patients with rheumatoid disease. Denosumab treatment led to a significant increase in BMD at the lumbar spine, total hip, femoral neck and trochanter. In addition, compared with placebo, denosumab treatment reduced the cartilage turnover marker C-telopeptide of type II collagen (CTx-II)/creatinine at 3 months, but not at 6 and 12 months.[16]

### Safety

Denosumab treatment was not discontinued due to safety reasons at any of the patients. One patient from the non-GCT group (3.3%) and three patients (10%) from the GCT group experienced fractures after the initiation of treatment. In general, denosumab was well tolerated by all patients and a good patient compliance and treatment adherence was observed.

## Conclusions

Our clinical data showed that 12-month treatment with denosumab increased BMD at lumbar spine and total hip, and reduced bone pain in women with OP regardless of its origin, postmenopausal or related to corticoid treatment. Both treatment groups achieved similar levels of increase in BMD and T-score of lumbar spine and total hip. Only, TBS appeared to be differently influenced by the treatment of the two groups, which comes as a confirmation of the suggestion that TBS represents an independent bone measure. The present data suggest that denosumab could be used as an effective and safe therapeutic option in patients not only with PMO but also with secondary OP related to long-term GCT.
